# Assessment of chicken peripheral blood mononuclear cells isolated from freshly drawn blood versus 24 h refrigerated blood

**DOI:** 10.14202/vetworld.2021.2549-2553

**Published:** 2021-09-27

**Authors:** Chananphat Tantikositruj, Anchalee Buadkhunthod, Jatuporn Rattanasrisomporn, Warangkana Kitpipit, Chaiwat Boonkaewwan

**Affiliations:** 1 Department of Animal Science, Faculty of Agriculture, Kasetsart University, Bangkok 10900, Thailand; 2 Department of Companion Animal Clinical Science, Faculty of Veterinary Medicine, Kasetsart University, Bangkok 10900, Thailand; 3 Akkhraratchakumari Veterinary College, Walailak University, Nakhon Si Thammarat 80161, Thailand; 4 One Health Research Center, Walailak University, Nakhon Si Thammarat, 80160, Thailand.

**Keywords:** Betong chicken, cell viability, leghorn chicken, lifespan, peripheral blood mononuclear cell

## Abstract

**Background and Aim:**

The peripheral blood mononuclear cell (PBMC) is an excellent cell source for *in vitro* studies, particularly those involving immunology. The aim of this study was to determine the quality and quantity of chicken PBMCs isolated from freshly drawn blood as well as blood that had been chilled for 24 h. In addition, the survival of PBMCs cultured in medium was investigated.

**Materials and Methods:**

Blood samples were collected from 12 Betong and 12 Leghorn chickens. Hemograms were analyzed. Density gradient centrifugation was used to isolate PBMCs. PBMCs (2×10^6^ cells/mL) were cultured in a culture medium and incubated in a CO_2_ incubator for 5 consecutive days. The number of viable cells was determined using the trypan blue dye exclusion method.

**Results:**

Blood samples were obtained from healthy chickens. There was no statistically significant difference in the total amount of PBMC between fresh and refrigerated blood samples from both chicken breeds. The viability of PBMCs isolated from fresh blood (95%) was significantly greater than blood refrigerated for 24 h (90-92%) in both breeds. Furthermore, the viability of PBMCs isolated from both blood samples decreased significantly over time, from 90-95% to 60-65%.

**Conclusion:**

The total number of PBMC in fresh and refrigerated blood was not significantly different. Fresh blood-derived PBMCs had significantly higher viability than 24 h refrigerated blood PBMCs. Furthermore, the viability of PBMCs decreased significantly over time.

## Introduction

The peripheral blood mononuclear cell (PBMC) population is composed primarily of lymphocytes (T cells, B cells, and NK cells), monocytes, and a small proportion of other immune cells such as dendritic cells [1]. PBMC preparations are frequently used in biomedical research, with applications ranging from simple cytotoxicity assays to single-cell functional and phenotypic immunological or molecular assays [2,3]. Thus, one can examine PBMCs to ascertain the immune response to specific stimuli. The most common method for isolating avian PBMCs is the centrifugal separation of blood components against a high-density medium [4]. Numerous factors can influence the quality of PBMCs, including the anticoagulant used, the rate at which blood is collected, the sample processing time, the technique used to obtain PBMCs, and their preservation [5]. These factors affect cell viability and cell count and thus can impair cell functions and potentially cause bias in the experimental results [6].

Numerous blood samples are delayed from processing due to the long distance or laboratory closures on weekends. Inaccurate, imprecise, and unreliable results may result from blood samples due to delays and poor storage [7,8]. Delays in obtaining adequate blood samples have been caused by various issues such as transport across long distances. When blood is stored for an extended period of time, significant time- and temperature-dependent changes can occur. It has been reported that basic parameters such as red blood cell (RBC) count, white blood cell (WBC) count, and platelet count remain stable for up to 24 h after collection with adequate storage (4°C-12°C). In addition, some measurements remain stable for up to 72 h after collection when refrigerated at 4°C [9,10].

PBMC is an excellent cell source for *in vitr*o studies, particularly immunology-related research [11-13]. Thus, to determine how to ensure high-quality PBMCs, we conducted this study to determine the quality and quantity of chicken PBMCs isolated from freshly drawn blood and blood that had been stored overnight at 4°C. In addition, the lifespan of PBMCs in a cultured medium was also investigated.

## Materials and Methods

### Ethical approval

All procedures used in this study were approved by Kasetsart University’s Animal Ethics Committee (ACKU61-AGR-009).

### Study period and location

The study was conducted from December 2018 to February 2020. Chickens for this study were provided by Kasetsart University’s Vajokkasikij Chicken Farm. The samples were processed at Department of Animal Science, Faculty of Agriculture and Department of Companion Animal Clinical Science, Faculty of Veterinary Medicine, Kasetsart University.

### Animals and blood collection

Whole blood was drawn from 12 Leghorn chickens (six males and six females) aged 14-16 weeks and 12 Betong chickens (six males and six females) aged 14-18 weeks. Blood samples (3.5 mL) were collected through the wing vein into an anticoagulant tube containing ethylenediaminetetraacetic acid. Each blood sample was segmented into three parts. The first portion (0.5 mL) was used to determine the blood hematology (complete blood count [CBC]). The second portion (1.5 mL) was used as a source of fresh blood for isolating PBMCs. The third portion (1.5 mL) was stored at 4°C overnight (24 h) before PBMC isolation.

### Hematological study

The first portion of the blood sample was analyzed for hematological abnormalities. Natt and Herrick’s method was used to determine the total number of RBCs and WBCs. A vet automated system (Sysmex 1000V, Norderstedt, Germany) was used to determine the hemoglobin (Hb) concentration. Hematocrit (Hct) was determined manually using microhematocrit capillary tubes. The differential WBC count was determined using monolayer blood films that had been fixed and stained with Wright’s stain.

### PBMC isolation and culture

The second and third portions of the fresh and 24 h blood samples were used to isolate PBMCs. Separation of PBMCs from blood samples was accomplished by gently layering 1.5 mL blood over 2 mL Histopaque® solution (Sigma-Aldrich, USA) and centrifuging at 1500 rpm for 30 min. The white band of mononuclear cells was harvested and washed 3 times with RPMI 1640 culture medium by centrifugation at 3000 rpm for 5 min. PBMCs were suspended in RPMI 1640 medium (containing 25 mM HEPES, 2 mM L-glutamine, 10% heat-inactivated fetal calf serum, penicillin [100 U/mL], and streptomycin [100 g/mL]) and then adjusted to a concentration of 2×10^6^ cells/mL. Then, 200 μL suspended PBMCs were seeded into a 96-well plate and incubated at 41°C in a humidified atmosphere containing 5% CO_2_ for 5 consecutive days.

### Cell viability test

A trypan blue dye exclusion test was used to determine cell viability. On the day of the cell viability test (day 0, day 1, day 2, day 3, day 4, and day 5), following a gentle mixing with pipetting, a volume of 10 μL of cultured PBMC was collected from each well.

### Statistical analysis

Prism 5 software (GraphPad Software, USA) was used to analyze the data, which are presented as the mean±standard deviation. The paired *t*-test was used to compare the viability and quantity of PBMCs isolated from freshly drawn blood to those of PBMCs isolated from 24 h blood refrigerated at 4°C. To determine the statistical significance of cell viability on days 0-5, one-way analysis of variance and Student–Newman–Keuls methods were used. p<0.05 was considered statistically significant.

## Results

### Hematological values

A CBC was conducted to ensure that blood samples were collected from healthy chickens. The hematological data are summarized in Table-1. The total erythrocyte count, Hb, and Hct of male and female Betong and Leghorn chickens were within the normal reference range. Male and female Betong and Leghorn chickens had normal total WBCs and differential leukocyte counts for heterophils, eosinophils, basophils, lymphocytes, and monocytes. This result indicated that the chickens’ blood samples were healthy.

**Table-1:** Hematological values of Betong (n=12) and Leghorn (n=12) chickens.

Hematological values	Betong		Leghorn	
	
	Male (n=6)	Female (n=6)	Male (n=6)	Female (n=6)
Red blood cell (10^6^/mL)	2.23±0.16	2.33±0.28	2.42±0.22	2.39±0.26
Hemoglobin (g/dL)	8.60±0.84	8.76±1.00	8.80±0.68	8.89±0.99
Hematocrit (%)	25.50±2.28	26.00±3.33	25.67±2.16	25.92±2.94
White blood cell (cells/mm^3^)	9570.00±1505.03	7874.17±2505.52	8891.67±1824.03	8039±2366
Heterophil (%)	66.83±7.39	66.33±8.58	65.17±7.83	65.67±5.76
Basophil (%)	0.00±0.00	0.25±0.62	1.00±1.67	0.67±1.30
Eosinophil (%)	1.83±1.33	1.92±1.83	0.67±1.21	1.08±1.31
Lymphocyte (%)	23.83±7.36	26.83±9.00	28.33±4.89	28.42±3.96
Monocyte (%)	3.33±1.21	3.42±1.08	4.00±0.63	3.67±1.50

Data are reported as mean±standard deviation

### Total amount of PBMC

Table-2 summarizes the total number of viable PBMCs isolated from 1.5 mL of fresh blood and 24 h refrigerated blood samples. The results indicate that there was no statistically significant difference in the total amount of PBMCs found in fresh and refrigerated blood samples from both chicken breeds. However, in both fresh and 24 h blood PBMCs, the average number of PBMCs in Betong chickens was significantly greater than in Leghorn chickens.

**Table-2:** Total peripheral blood mononuclear cells isolated from 1.5 mL blood samples taken from Betong and Leghorn chickens.

Chicken	Total PBMC	(×10^6^)
	
	Fresh blood	24 h blood
Betong		
Male (n=6)	4.770±0.438	4.752±0.161
Female (n=6)	5.079±0.420	4.943±0.334
Average	4.925±0.358^a^	4.847±0.296^a^
Leghorn		
Male (n=6)	4.566±0.278	4.427±0.216
Female (n=6)	4.059±0.170	3.995±0.169
Average	4.312±0.344^b^	4.211±0.291^b^

Data are reported as mean±standard deviation. ^a, b^Each column and row are assigned a different letter to denote their significance (p<0.05). PBMC=Peripheral blood mononuclear cell

### PBMC cell viability

Tables-3 and 4 present cell viability data from Betong and Leghorn chickens, respectively, to illustrate the percentage of viable cells in the PBMC suspension. The average cell viability of PBMC isolated from fresh blood versus 24 h blood samples of Betong chicken was compared from day 0 to day 5. The results indicated that PBMC isolated from fresh blood has significantly higher viability than PBMCs isolated from 24 h blood. In addition, the viability of PBMC from Betong chicken in both blood samples decreased significantly over time, from 92-95% on day 0 to 63-65% on day 5. PBMCs from Leghorn chickens produced a similar result. Fresh blood PBMC has significantly higher viability than 24 h blood PBMC on days 1-5 in Leghorn chickens. In addition, the viability of PBMCs in both blood samples decreased significantly over time, from 90-95% on day 0 to 60-65% on day 5.

**Table-3:** The viability of peripheral blood mononuclear cells isolated from fresh and 24 h blood samples from Betong chickens (n=12).

Time	Cell viability (%)					

	Fresh blood			24 h blood		
	
	Male	Female	Average	Male	Female	Average
Day 0	94.89±0.90^a^	95.86±1.05^a^	95.37±1.05^a^	93.17±1.18^A^	92.29±1.34^A^	92.73±1.29^A^
Day 1	91.89±0.81^b^	92.81±1.16^b^	92.32±1.08^b^	90.17±1.08^B^	89.08±1.28^B^	89.62±1.30^B^
Day 2	87.64±0.71^c^	88.83±1.43^c^	88.21±1.25^c^	85.92±1.07^C^	85.19±1.33^C^	85.55±1.25^C^
Day 3	82.23±0.63^d^	83.66±1.60^d^	82.91±1.39^d^	80.51±1.11^D^	80.3±1.39^D^	80.40±1.24^D^
Day 4	74.73±0.75^e^	76.68±1.55^e^	75.67±1.56^e^	73.01±1.26^E^	73.17±1.19^E^	73.08±1.22^E^
Day 5	64.74±1.26^f^	66.43±1.47^f^	65.56±1.59^f^	63.02±1.68^F^	62.98±1.08^F^	63.00±1.43^F^

Data are reported as mean±standard deviation. ^a, b, c, d, e, f^ and ^A, B, C, D, E, F^: The distinction between capital and lowercase letters indicated the significance of each column and each row’s significant differences are denoted by a different letter (p<0.05)

**Table-4:** The viability of peripheral blood mononuclear cells isolated from fresh and 24 h blood samples from Leghorn chickens (n=12).

Time	Cell viability (%)					

	Fresh blood			24 h blood		
	Male	Female	Average	Male	Female	Average
Day 0	94.96±0.73^a^	95.32±1.02^a^	95.14±0.86^a^	90.91±0.95^A^	91.05±0.99^A^	90.98±0.92^A^
Day 1	91.91±0.74^b^	91.90±1.16^b^	91.90±0.92^b^	87.86±0.94^B^	87.98±0.98^B^	87.92±0.92^B^
Day 2	87.66±0.77^c^	88.35±1.05^c^	88.01±0.95^c^	83.61±1.02^C^	83.88±1.14^C^	83.75±1.04^C^
Day 3	82.24±0.90^d^	82.89±1.00^d^	82.56±0.96^d^	78.20±1.24^D^	78.30±0.84^D^	78.25±1.01^D^
Day 4	74.78±1.15^e^	76.07±0.89^e^	75.42±1.19^e^	70.70±1.41^E^	71.79±0.98^E^	71.24±1.29^E^
Day 5	64.79±1.60^f^	65.96±0.97^f^	65.37±1.47^f^	60.71±1.90^F^	60.66±1.00^F^	60.69±1.45^F^

Data are reported as mean±standard deviation. ^a, b, c, d, e, f^ and ^A, B, C, D, E, F^: The distinction between capital and lowercase letters indicated the significance of each column and each row’s significant differences are denoted by a different letter (p<0.05)

## Discussion

Numerous chicken breeds have been developed for a variety of purposes. The current study used the chickens of both meat and laying breeds. The Betong chicken is popular chicken meat in Thailand’s southern region due to its superior meat quality, low carcass fat content, and high lean meat content [14]. White Leghorns are widely used as layer chickens in a variety of countries. They are the industry standard white egg producers due to their high efficiency in feed conversion [15]. Hematological values are critical in chickens; they serve as the primary tool for determining chickens’ health statuses [16]. The hematological parameters of 12 Betong and 12 Leghorn chickens used in this study were within the normal range, which indicated that blood samples were obtained from healthy chickens.

The scientific community has long held the belief that “old blood samples” are unsuitable for isolating PBMCs for functional assays. A CBC is the most frequently performed laboratory test, as it provides critical information [17]. It has been reported that blood samples for CBC can be safely stored for 24 h. Refrigeration (at 4°C) would be a better option for extended storage [18]. Furthermore, WBC, RBCs, Hb, neutrophils, and lymphocytes remained stable for up to 48 h [19]. PBMCs have been used extensively in a variety of fields of research, including immunology [3,11-13]. Our study established that there is no statistically significant difference in the amount of PBMCs found in fresh blood samples and 24 h blood samples stored overnight at 4°C from both chicken breeds. However, the difference in the number of PBMCs between Betong and Leghorn in both fresh and 24 h blood PBMC may be due to the breed differences; Betong is a meat type chicken, whereas Leghorn is a laying type chicken [14,15].

The use of trypan blue dye exclusion staining to determine cell viability is a time-honored technique. This procedure has been used in academic research laboratories and industrial biotechnology plants to determine the number of viable cells present in a cell suspension on the basis that live cells have intact cell membranes that are resistant to trypan blue dye. Before conducting an *in vitro* study, it is critical to determine cell viability, which serves as an early indicator of cell quality; viability values greater than or equal to 95% are considered to have excellent quality [20].

It has been reported that a 24 h delay in the processing of blood samples had no effect on the viability of PBMCs [21]. Regrettably, our study discovered that PBMCs isolated from fresh blood have significantly higher viability (day 0: 95.37% and 95.14%) than PBMCs isolated from 24 h refrigerated blood (day 0: 92.73% and 90.98%). Previous research has examined the effect of sample processing delays on a single immunologic parameter after a 24 h period, and the results indicated a decrease in cellular viability after 24 h, which could have significant biological implications. The average viability of 92% decreased significantly to 84% when processing was delayed for 24 h [22]. Even though this study discovered that 24 h blood had lower cell viability than fresh blood, it was still greater than 90%, which indicated that the cells were still of good quality.

The current study established that both fresh and 24 h refrigerated blood PBMC viability in cultured medium decreased significantly over time. At the start, middle, and end of the experiment, viability was approximately 90-95%, 78-82%, and 60-65%, respectively. Three days should be sufficient for maintaining PBMCs at 80% viability in a culture medium. Due to the limited viability of PBMCs in culture, PBMCs must be processed and cryopreserved [23]. However, it has been well established that significant cell loss occurs during cryopreservation and thawing, most likely as a result of freezing-induced stress and cell loss associated with additional washing and aspiration steps [24].

## Conclusion

Blood samples were collected from healthy meat (Betong) and laying (Leghorn) chickens. There was no statistically significant difference in the total number of PBMCs of fresh and 24 h refrigerated blood. Although fresh blood PBMCs are significantly more viable than 24 h refrigerated blood PBMCs, the viability of PBMCs isolated from both blood samples was greater than 90%. Furthermore, the viability of PBMCs isolated from both blood samples decreased significantly over time.

## Authors’ Contributions

CT and AB: Collected the samples and conducted the experiments. JR: Instrumental in assisting with the technical aspects of the experiments. WK: Data analysis. CB: Conceived the experiments, collected data, and edited the manuscript. All authors read and approved the final manuscript.
